# It is challenging to reproduce both anatomical and functional aspects of anterolateral reconstruction: postoperative 3D-CT analysis of the femoral tunnel position

**DOI:** 10.1186/s43019-024-00230-9

**Published:** 2024-08-29

**Authors:** Dong Jin Ryu, Seoyeong Kim, Minji Kim, Joo Hwan Kim, Won Jae Kim, Dohyung Lim, Joon Ho Wang

**Affiliations:** 1 Department of Orthopaedic Surgery, Inha University Hospital, Inha University School of Medicine, Incheon, South Korea; 2 Department of Mechanical Engineering, Sejong University, Seoul, South Korea; 3 RNX Co., Ltd, Seoul, South Korea; 4 Department of Orthopaedic Surgery, Samsung Medical Center, Sungkyunkwan University School of Medicine, Seoul, 06351 South Korea; 5 Department of Health Sciences and Technology, SAIHST, Sungkyunkwan University, Seoul, 06351 South Korea; 6 Department of Medical Device Management and Research, SAIHST, Sungkyunkwan University, Seoul, 06351 South Korea

**Keywords:** Anterolateral ligament, Femoral tunnel position, Anatomy, Reliability

## Abstract

**Background:**

This study aimed to evaluate the femoral tunnel position and fiber length of the anterolateral ligament (ALL) reconstruction compared with the natural anatomy of the ALL. We also evaluated whether the femoral tunnel position would affect residual pivot shift.

**Methods:**

This study was a retrospective review of 55 knees that underwent ALL reconstruction considering the anatomical and functional aspects, during primary anterior cruciate ligament (ACL) reconstruction in the presence of a high-grade pivot shift or revisional ACL reconstruction. We determined the position of the femoral tunnel and the length of graft using a three-dimensional (3D)-computed tomography (CT) model after ALL reconstruction. We also measured graft excursion during surgery and examined pivot shift 2 years after surgery. We conducted a subgroup analysis of femoral tunnel position, fiber length, isometricity, and residual pivot shift depending on whether the tunnel was anterior or posterior to the lateral epicondyle (LE). We also performed a subgroup analysis depending on whether the ACL reconstruction was primary or revisional.

**Results:**

The mean femoral tunnel position was 2.04 mm posterior and 14.5 mm proximal from the center of the LE. The mean lengths of the anterior and posterior fibers were 66.6 and 63.4 mm, respectively. The femoral tunnel was positioned more proximally than the anatomical position, and both anterior and posterior ALL fibers were longer than the natural anatomy. The anteroposterior femoral tunnel position was significantly correlated with anterior (*p* = 0.045) and posterior (*p* = 0.037) fiber excursion. In the subgroup analysis, there was no significant difference in the residual pivot shift between the posterior and anterior tunnel positions. However, there were significant differences for proximal position (*p* < 0.001) and fiber length (*p* = 0.006). There was no significant difference between primary and revisional ACL regarding femoral tunnel position and fiber lengths.

**Conclusion:**

It is challenging to reproduce both anatomical and functional aspects of ALL reconstruction in both primary and revision ACL reconstruction. Especially for functional reconstruction, the femoral tunnel tended to be positioned more proximally than the anatomical position. However, the femoral tunnel position did not affect functional clinical outcomes at the 2-year follow-up.

**Level of evidence:**

Level IV Case series.

## Background

The reconstruction of the anterolateral ligament (ALL) has been increasingly debated in the recent literature. Following its description by Segond in 1879, the ALL has been investigated in many anatomical studies [1–8]. Some biomechanical studies have shown the role of the ALL in the rotational stability of the knee, with disruption in stability revealed by the pivot shift [9–12]. On the basis of exploratory cadaveric testing, many researchers agree that the ALL is an important stabilizer of internal tibial rotation. Irrespective of the operative technique, a high rate of residual pivot shift remains after anterior cruciate ligament (ACL) reconstruction [13–15]. This has encouraged surgeons to focus on additional procedures for rotational instability, particularly for anterolateral structures such as the ALL.

Previous studies have introduced ALL reconstruction with the formation of femoral tunnel and insertion of the ALL [16–21]. Although there were inconsistencies in the femoral tunnel position, recent research has shown that the femoral tunnel position is located posterior and proximal to the lateral epicondyle (LE), with the length of the ALL varying from 30.41 to 59.0 mm in anatomical studies [22]. However, some studies still report that the anterior to the LE location is more advantageous for restoring isometry [21].

Considering the biomechanical effect of the ALL [23], a functional femoral tunnel position is essential to reduce anterolateral rotational instability (ALRI), with a range of 0–30° flexion. However, similar to that in anatomical ACL reconstruction [24, 25], it is difficult to consistently reproduce the optimal position. Imbert et al. [26] reported that proximal and posterior to the LE is the only position with a favorable isometry, being tight in extension and in internal rotation at 20°. The non-anatomical nature of ALL reconstruction has the potential risk of over-constraint and changes in knee biomechanics [27].

To our knowledge, no study has the investigated the consistency and accuracy of the femoral tunnel position after ALL reconstruction. This study aimed to evaluate the femoral tunnel position and the fiber length of the ALL compared with the ALL natural anatomy. Moreover, we evaluated whether the femoral tunnel position would affect the residual pivot shift.

## Methods

### Subjects

This study was a retrospective review of 59 knees in 59 patients who underwent ALL reconstruction between July 2019 and February 2020 during primary ACL reconstruction in the presence of a high-grade pivot shift [≥ International Knee Documentation Committee (IKDC) grade 2] or during revisional ACL reconstruction. The inclusion criteria were as follows: (1) age 18–60 years; (2) confirmed ACL injury with pivot shift of IKDC grade ≥ 2; (3) revisional ACL reconstruction, and (4) follow-up for at least 2 years after surgery. The exclusion criteria were as follows: (1) accompanied other ligament injury (*n* = 3) and (2) a follow-up period less than 2 years after surgery (*n* = 1).

Finally, 55 patients (55 knees) were enrolled in this study. This study was approved by the ethics committee of our medical center and received institutional review board approval (IRB No. SMC 2020-12-099).

### Surgical procedure

After primary single-bundle ACL reconstruction or revision, we performed ALL reconstruction as described previously [19, 28, 29]. We used the auto-gracilis tendon for ALL reconstruction in patients undergoing primary ACL surgery. For revision surgery, we used the remnant side of the allograft from the allo-tibialis or allo-Achilles tendon after preparing the ACL graft. The ALL graft 4.5 mm in diameter was prepared. All surgical procedures were performed by the one senior expert knee surgeon (J.H.W).

**Fig. 1.**
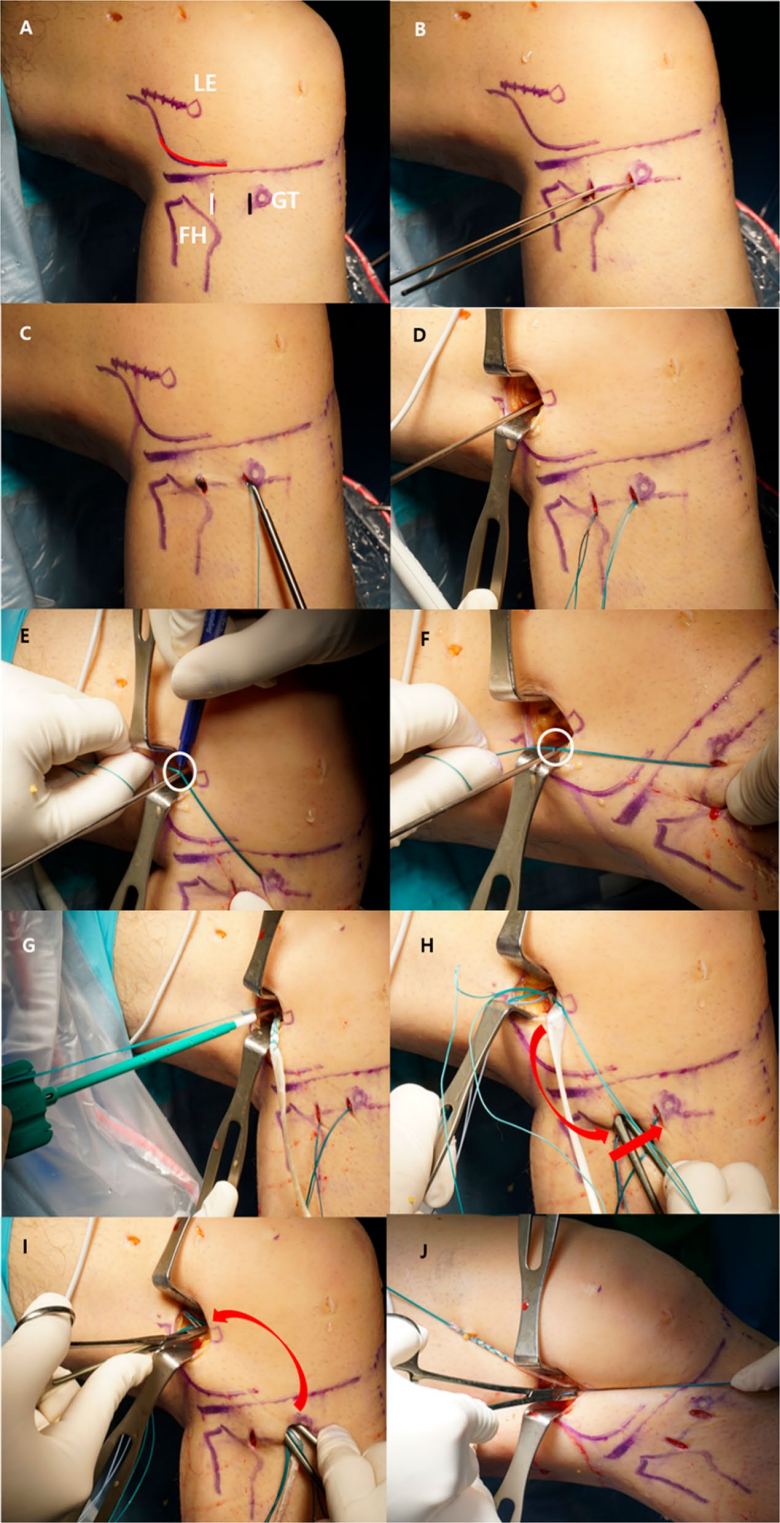
The procedure of the anterolateral ligament reconstruction, right knee. **A** Three bony landmarks are marked 1) the lateral epicondyle (LE) of the femur (white circle) and the inferolateral border of the lateral condyle (red line), 2) the head of the fibula, and 3) Gerdy’s tubercle. A 1 cm-sized mini-incision is made just above Gerdy’s tubercle’s superoposterior margin (black line). A second small incision is made at the midpoint between the fibular head and Gerdy’s tubercle (white line). **B** Two guide K-wires inserted at the target points. **C** After reaming approximately 20 mm with a 4.5 mm diameter reamer, a loop passer is introduced through the anterior tunnel to the posterior tunnel. **D** A 1.5 cm sized stab incision is made at just proximal and posterior to the LE, and a guide K-wire is inserted. **E**, **F** Excursions are checked. After the fiber length is marked using an Ehibond (white circle), the knee is moved from 90° flexion to extension, and we measure the length of the anterior fiber (white circle) and posterior fiber. **G** When acceptable excursion is confirmed, a 20 mm deep femoral tunnel is drilled using a 4.5 mm reamer. The graft is then placed in the femoral tunnel with the anchor. **H** A suture grasper is passed through the lateral stab incision from the posterior tibial tunnel to the proximal femoral side, deep to the fibers of the ITB, and the graft is retracted. The graft is then passed to the anterior side through the tibial trans-osseous tunnel using the Ethibond suture relay. I. The end of ALL graft is retracted on the proximal femur side, held by the suture grasper under the ITB once again. J. To secure the ALL graft, the knee is fully extended with neutral foot rotation. The graft end is tied to the femoral side with an anchor attached to the fiberwire. *LE* lateral epicondyle; *FH* fibular head; *GT* Gerdy’s tubercle; *ITB* Iliotibial band; white circle denotes the excursion, and the red arrow indicated the passing of the graft

Three bony landmarks were marked as follows: (1) LE of the femur, (2) head of the fibula, and (3) Gerdy’s tubercle (Fig. 1A). The femoral anatomical points were selected using the method suggested by Sonnery-Cottet et al. [19]. A 1 cm-sized mini incision was made just above the superolateral margin of Gerdy’s tubercle (α). A second small incision was made at the midpoint between the fibular head and Gerdy’s tubercle, which was taken as the point of attachment of the native ALL (β; Fig. 1A, B) [2]. Two K-wires were inserted into the bone at the two selected points. After reaming over the inserted guide K-wire for approximately 20 mm with a reamer measuring 4.5 mm in diameter, a loop passer was introduced through the tibial anterior tunnel to the posterior tunnel (Fig. 1C). No.2 Ethibond was introduced using the loop passer, and a loop was made at the posterior tibial tunnel. A third 1.5 cm-sized stab incision was made just proximal and posterior to the LE. A K-wire was inserted into the bone just proximal and posterior to the LE (γ; Fig. 1D). The knee was then moved from 90° flexion to extension, and we measured the length of the anterior fiber [between (γ) and (α) point] and posterior fiber [between (γ) and (β)] using the applied Ethibond and a ruler (Fig. 1E, F).

We selected a point that slightly increased the fiber length with extension and decreased with flexion to 90°. This “excursion” meant that the graft would be tighter in extension and lax as the knee was flexed [30]. If the fiber length increased during knee flexion (graft tightened in flexion), we repositioned the femoral side K-wire in the proximal, posterior, or both directions. After confirming the excursion of the anterior and posterior fibers were acceptable, we drilled a 20 mm-deep femoral tunnel using a 4.5 mm-wide reamer. The graft was then placed into the femoral tunnel with a 4.75 mm anchor (SwiveLock®, Arthrex, Naples, Florida, USA; Fig. 1G).

A suture grasper was passed through the lateral stab incision from the posterior tibial tunnel to the proximal side of femur, deep into the fibers of the iliotibial band (ITB; Fig. 1H). The end of the proximal fixated ALL graft was retrieved by the suture grasper to the posterior side of the tibial tunnel and passed to the anterior tibial tunnel through the intraosseous tunnel using a prepared Ethibond loop. Finally, the end of the ALL graft was retracted to the proximal femur side by a suture grasper under the ITB again (Fig. 1I). To secure the ALL graft, the knee was fully extended with the foot in neutral rotation [30].

The graft end was tied at the femoral side with the fiberwire pre-attached anchor (Fig. 1J).

### Criteria for determining femoral tunnel location considering ALL function

To reduce the tibia’s rotational force, tensile force was applied to the ALL graft during the range of 0 ~ 45° knee joint angle, and consequently, the length of the ALL graft was increased. When the knee is flexed more than 90°, an over-strain may be applied to the lateral compartment if tension is applied to the ALL graft [30]. The “excursion” is the increasing the length of ALL fiber during 90° flexion to extension of knee joint. We adjusted it several times to find the optimal position and recorded the adjustment number.

If the femoral tunnel was positioned within the proximal and posterior boundary of LE, we defined it as “anatomical ALL.” However, if the femoral tunnel was positioned after several adjustments considering the excursion and function, we described it as “functional ALL.”

### Rehabilitation

All patients were sent to our sports medical rehabilitation department and were rehabilitated using the same protocol by physical therapists as previously described [31, 32]. For 4 weeks after surgery, partial weight-bearing ambulation with crutches was allowed. Full weight-bearing walking was permitted at 6 weeks. The range of motion with a brace gradually increased from 2 days after surgery and reached 120° knee flexion by 6 weeks. If patients underwent concomitant meniscal repair, the knee was immobilized for 2 weeks after surgery. Quadriceps sets and ankle pump exercise were started on the first postoperative day. Closed kinetic chain exercises were initiated 2 weeks postoperatively and return to sports was allowed after 9 months.

### Measurement of the femoral tunnel position and fiber length using 3D-CT reconstruction

**Fig. 2.**
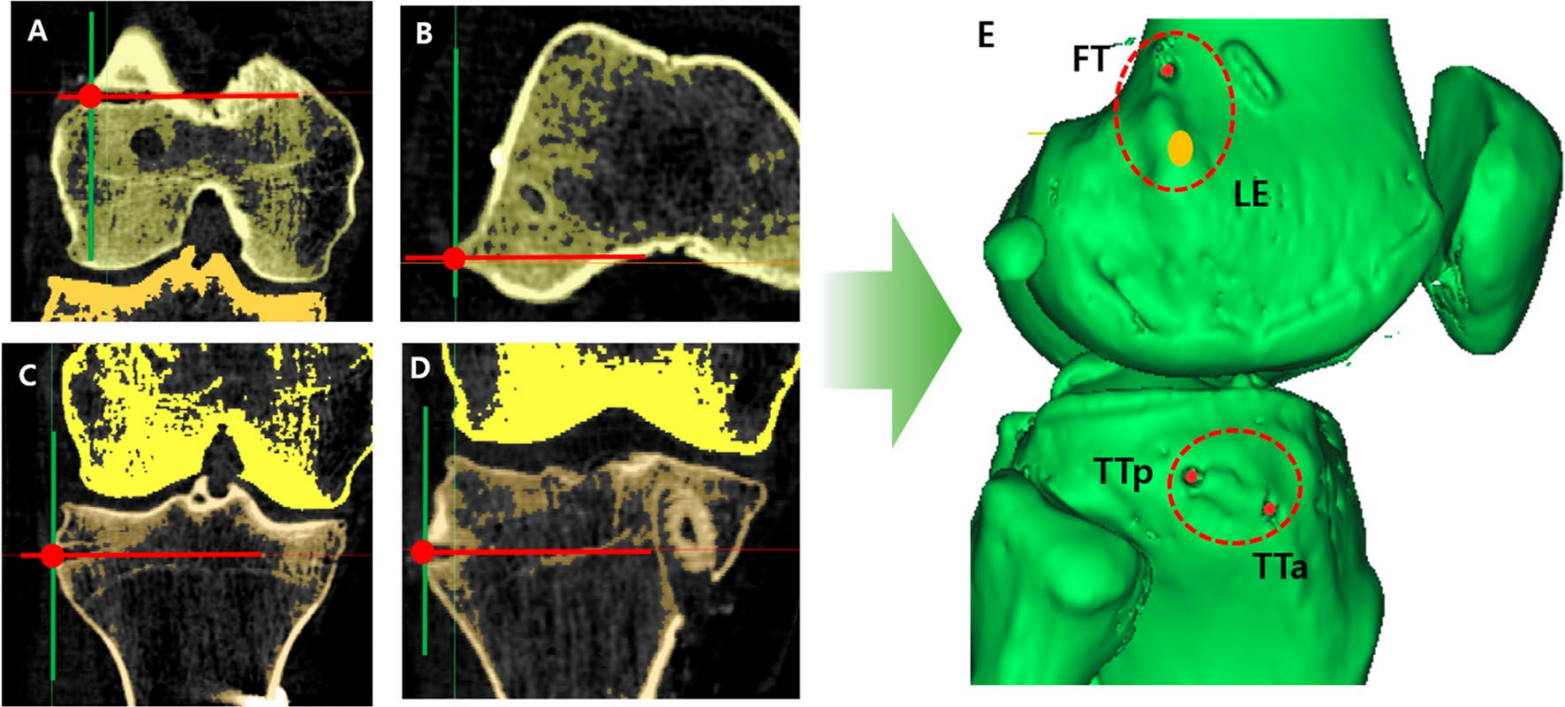
3D CT reconstruction and setting of the center points of each landmark. The center point (red circle) at the **A** femoral tunnel (FT), **B** lateral epicondyle (LE) of the femur, **C** posterior tunnel of the tibia (TTp), **D** anterior tunnel of the tibia (TTa), and **E** 3D-reconstructed image developed by setting the center point of the LE (yellow circle)

Two days after surgery, the negative suction drain was removed, and the patients underwent computed tomography (CT) to assess the ACL and ALL tunnel positions. After three-dimensional (3D)-CT reconstruction, we selected a point at the intersection of the center axis of the tunnel and the expected position of the cortical bone (Fig. 2A–D). Thereafter, the point was marked on the 3D image, and the center point of the LE was set (Fig. 2B).

**Fig. 3.**
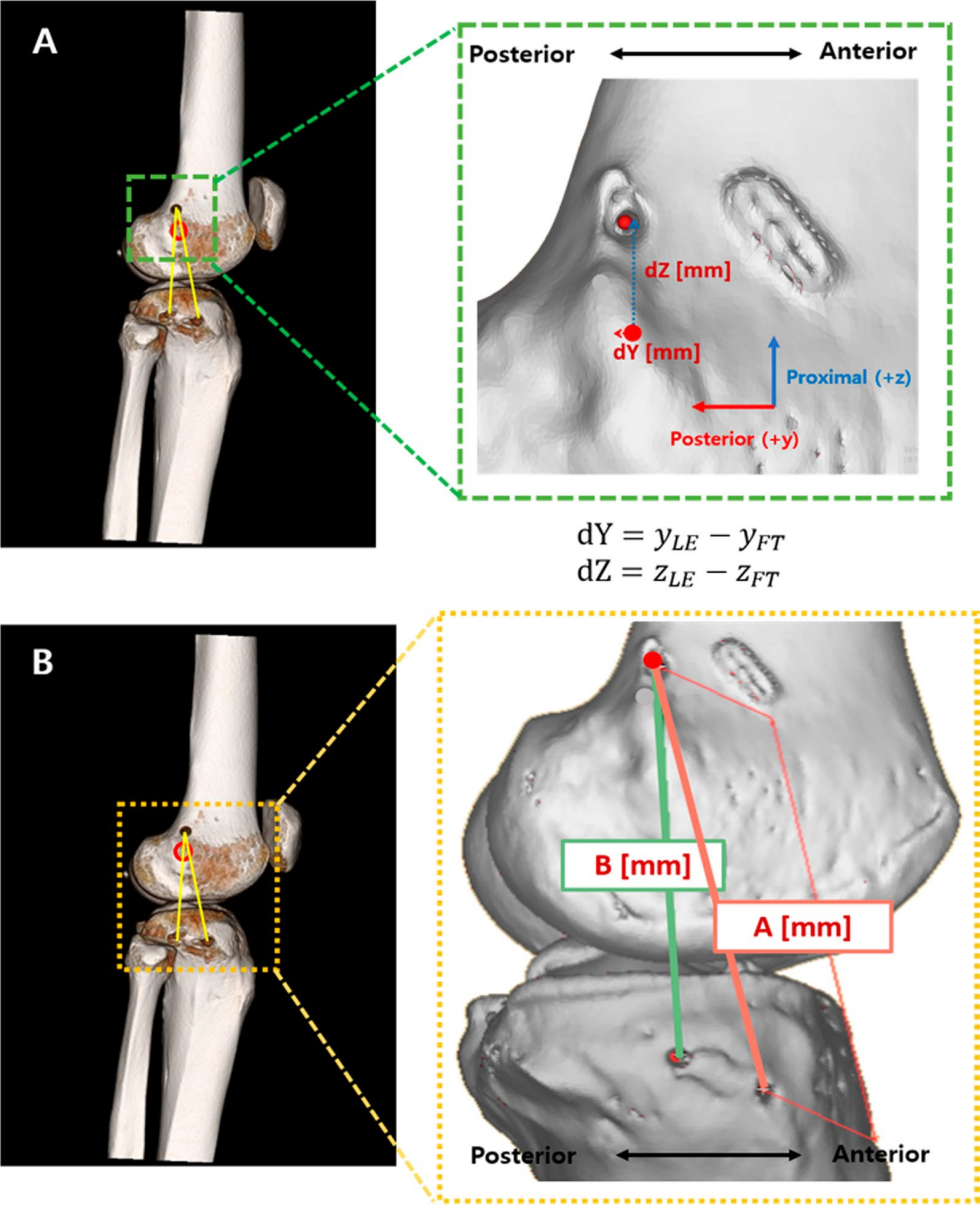
Calculation of the femur tunnel position based on the lateral epicondyle. **A** Femoral tunnel position from the center point of the lateral epicondyle. **B** The length of the anterior and posterior fibers. *FT Femoral tunnel, *LE* lateral epicondyle, *TTp* posterior tunnel of the tibia, *TTa* anterior tunnel of the tibia

The position of the ALL femoral tunnel was determined on the basis of the LE (Fig. 3). The anteroposterior (dY) and proximal–distal (dZ) lengths were measured (Fig. 3A). For fiber length, the anterior fiber (A) and posterior fiber (B) lengths were measured using the following equation, considering the 3D structure (Fig. 3B). These 3D-CT measurements were performed by two independent investigators (S.K. and M.K.).

### Subgroup analysis

Considering the recent consensus on the femoral tunnel position, we performed subgroup analysis 1 following the femoral tunnel position (anterior or posterior to LE). We compared the fiber length, excursion, isometricity, residual pivot shift, and clinical outcomes including Lysholm score and IKDC subjective score at 2-year follow-up.

We also performed subgroup analysis 2 with primary or revisional ACL. Revisional ACL requires technical considerations to determine the ideal femoral ALL tunnel position. We compared the femoral tunnel position, fiber length, excursion, and a number of adjustments (positioning error). We also examined the tunnel location and subsequent treatment records of patients who remained with high-grade rotational instability, representing more than pivot shift grade 2.

### Statistical analysis

Pearson correlation analysis was used to confirm the correlation between the femoral tunnel position and anterior or posterior fiber excursion. The independent *t*-test and Fisher’s exact test were used for the subgroup analyses regarding the anterior and posterior positions. Statistical significance was defined as *p* < 0.05. Two independent investigators measured the parameters at intervals of 3 weeks between the measurements. The intraclass correlation coefficient (ICC) was used to determine intra- and inter-observer reliability, and ICC > 0.8 was considered to indicate good reliability. Average values of the measurements were used in the analysis. All parameters showed good correlation (> 0.86).

G-power (version 3.1, Institut für Experimentelle Psychologie, Heinrich Heine Universität, Dusseldorf, Germany) was used to perform a post-hoc power calculation to compare the anterior and posterior positions [33]. Based on the sample size of this study and an α-error of 0.05, there was adequate power (0.93) to detect a significant difference between the two groups.

## Results

**Fig. 4.**
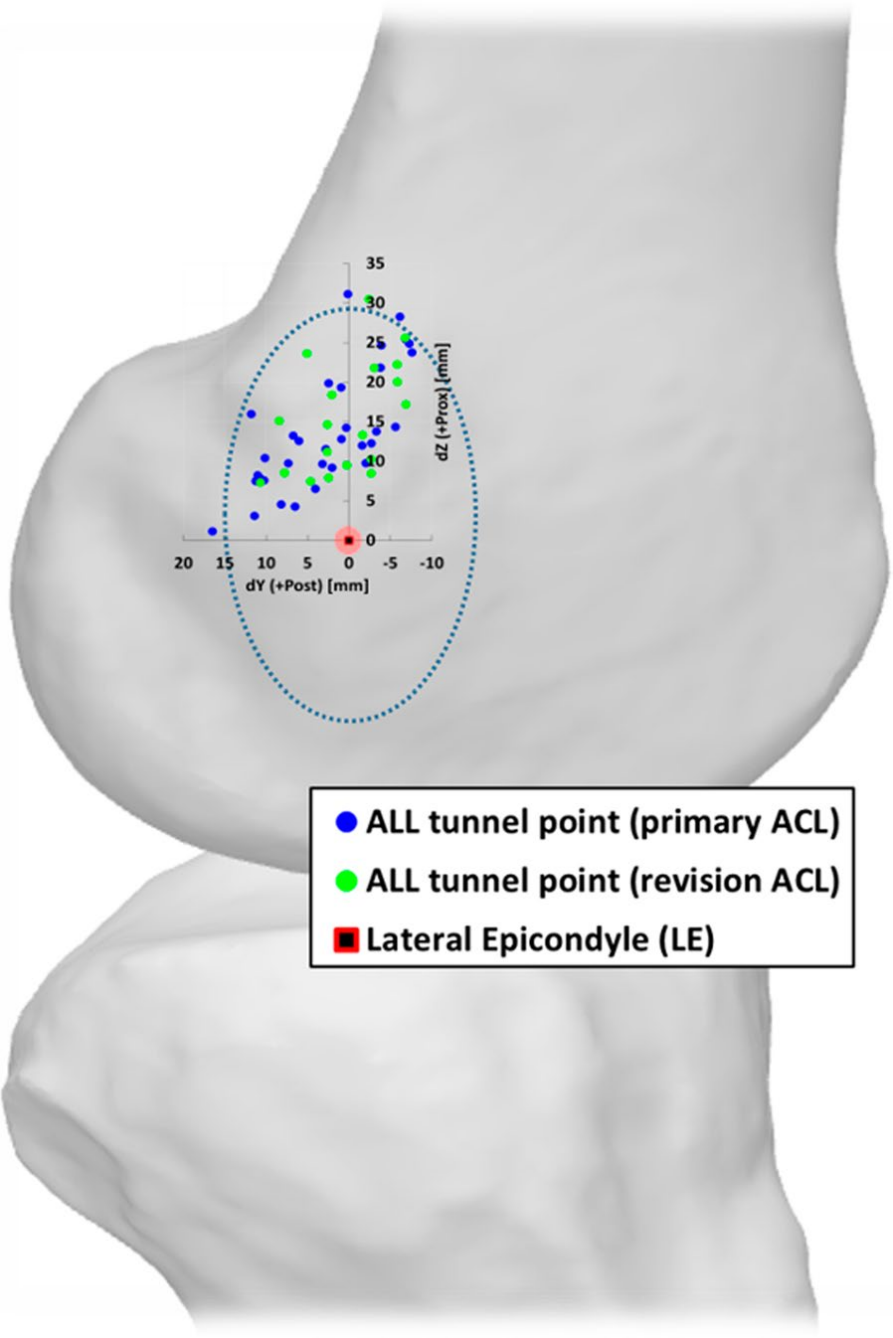
ALL femoral tunnel positions are based on the center point of the lateral epicondyle. (Blue dot circle: boundary of the lateral epicondyle)

The patient demographics are summarized in Table 1. The mean femoral tunnel position of the ALL was 2.04 mm (± 6.6 mm) posterior, and 14.5 mm (± 8.2 mm) proximal from the center point of the LE. The femoral tunnel was positioned more proximally than the anatomical position. Although 20 cases (36.3%) involved the anterior positioning of the ALL relative to the center of the LE (average, 4.4 mm), these were mostly included in the LE boundary (Fig. 4). In contrast, there was no case of distal positioning relative to the center of the LE. The mean length of the anterior ALL fiber was 66.6 mm (± 8.8 mm) and that of the posterior ALL fiber was 63.4 mm (± 8.7 mm). Compared with ALL natural anatomy (30.41 ~ 59 mm), both anterior and posterior fibers were measured to be longer.

**Table 1:** Patient demographics

Variables	Mean ± SD or number
Sex (male/female)	44:11
Age, years	26.8 ± 9.5
Height, cm	172.4 ± 8.0
Weight, kg	77.9 ± 12.5
Body mass index, kg/m^2^	26.1 ± 3.4
Preoperative pivot shift (IKDC)	
0 (equal)	0
1 (glide)	0
2 (clunk)	10 (18.2%)
3 (gross)	45 (81.8%)
Primary ACL: revision ACL, n	35:20

*SD* standard deviation, *IKDC* International Knee Documentation Committee, *ACL* anterior cruciate ligament

With these femoral tunnel positions, the excursion of the anterior fiber was 2.6 ± 3.0 mm from 90° flexion to extension. For the posterior fiber, the excursion was 6.9 ± 3.8 mm. Pearson correlation analysis showed that the anteroposterior femoral position was significantly correlated with anterior (*r* = 0.276, *p* = 0.045) and posterior (*r* = 0.287, *p* = 0.037) excursion. The posterior femoral position was positively correlated with excursion. However, there was no significant correlation between the proximal–distal tunnel position and anterior (*r* = −0.146, *p* = 0.296), and posterior excursion (*r* = −0.017, *p* = 0.902).

In subgroup analysis 1, there was no difference in residual pivot shift (rotational instability) at the 2-year follow-up regardless of the femoral anteroposterior tunnel position (Table 2). However, there were significant differences in the femoral tunnel position (proximal–distal), and in the anterior and posterior fiber lengths. The mean number of adjustments required to identify the ideal point was 1.8 for the posterior position and 3.1 for the anterior position, with a significant difference between the two groups (*p* < 0.001). However, there was no difference in functional outcome, representing Lysholm and IKDC subjective score, at the 2-year follow-up.

**Table 2:** Subgroup analysis of the posterior and anterior position groups

Variables	*Posterior* position (*n* = 35)	*Anterior* position (*n* = 20)	*p*-Value
Sex (male/female), *n*	29:5	15:6	0.183
Age, years	25.8 ± 9.0	28.6 ± 10.4	0.313
Height, cm	172.4 ± 8.1	172.4 ± 8.2	0.999
Weight, kg	78.4 ± 13.1	77.2 ± 11.6	0.716
Body mass index	26.3 ± 3.7	25.8 ± 2.9	0.595
Femur tunnel (anteroposterior)^†^, mm	5.8 ± 5.2	−4.4 ± 1.98	** < 0.001**
Femur tunnel (proxi-distal)^‡^, mm	11.2 ± 6.3	20.5 ± 8.1	** < 0.001**
Anterior fiber length, mm	63.9 ± 6.9	71.4 ± 9.9	**0.006**
Posterior fiber length, mm	60.1 ± 6.4	69.4 ± 9.3	** < 0.001**
Anterior fiber excursion, mm	3.0 ± 3.3	2.0 ± 2.3	0.153
Posterior fiber excursion, mm	7.5 ± 4.0	6.0 ± 3.4	0.136
Anterior fiber isometric, *n*	24	13	0.507
Posteriorfiber isometric, *n*	5	4	0.423
Both isometric, *n*	5	3	0.617
Number of adjustments, *n*	1.8 ± 0.8	3.1 ± 1.1	** < 0.001**
Pivot shift (IKDC) at 2 years, *n*			
0 (equal)	0	0	1.0
1 (glide)	0	1 (5%)	
2 (clunk)	1 (2.8%)	1 (5%)	
3 (gross)	1 (2.8%)	0	
Lysholm score at 2 years	85.8 ± 15.1	82.2 ± 15.0	0.767
IKDC subjective score at 2 years	83.1 ± 13.6	80.8 ± 16.8	0.374

The data are presented as mean ± SD or as *n* (%)

^†^Positive value: posterior to the center of the lateral epicondyle

^‡^Positive value: proximal to the center of the lateral epicondyle

*IKDC* International Knee Documentation Committee

In subgroup analysis 2, 35 knees underwent primary ACL reconstruction, and 20 knees underwent revisional ACL reconstruction (Table 3). There was no significant difference between the two groups in the femoral tunnel position, anterior fiber excursion, posterior fiber excursion, or fiber length. However, the number of adjustments was higher in the revisional ACL group (*p* = 0.039).

**Table 3:** Subgroup analysis for primary and revisional anterior cruciate ligament reconstruction

Variables	Primary ACL (*n* = 35)	Revision ACL (*n* = 20)	*p*-Value
Sex (male/female), n	28:7	16:4	0.643
Age, years	25.3 ± 9.8	29.6 ± 8.5	0.095
Height, cm	172.27 ± 7.75	172.74 ± 8.81	0.842
Weight,kg	79.2 ± 14.3	75.8 ± 8.5	0.338
Body mass index	26.6 ± 3.9	25.4 ± 1.9	0.208
Femur tunnel (anteroposterior)^†^, mm	3.16 ± 7.22	0.8 ± 4.93	0.099
Femur tunnel (proxi-distal)^‡^, mm	13.38 ± 7.77	16.7 ± 8.9	0.154
Anterior fiber length, mm	66.33 ± 7.60	67.13 ± 10.95	0.750
Posterior fiber length, mm	63.13 ± 7.88	64.09 ± 10.36	0.721
Anterior fiber excursion, mm	2.37 ± 2.77	3.15 ± 3.39	0.389
Posterior fiber excursion, mm	6.74 ± 3.55	7.4 ± 4.34	0.569
Number of adjustments, *n*	2.09 ± 0.91	2.70 ± 1.21	0.039

^†^Positive value: posterior to the lateral epicondyle center

^‡^Positive value: proximal to the lateral epicondyle center

*ACL* anterior cruciate ligament reconstruction

There were three cases of residual high-grade pivot shift during the 2 years after surgery. Two patients had reinjury by slip down and confirmed re-tear of the primary ACL graft at 6 and 7 months after surgery, respectively. One was in the posterior position group, and the other was in the anterior position group. The patients underwent revisional ACL surgery at 9 and 11 months after surgery, respectively. One patient in the posterior position group who performed revisional ACL did not report any trauma; however, residual grade 2 pivot shift was noted 2 years after surgery. The patient did not experience any discomfort or instability; thus, conservative treatment was employed.

## Discussion

The most important finding of this study was that it was difficult to satisfy both the anatomical and functional aspects of ALL femoral tunnel formation with high reproducibility. We tried first to set the anatomical position and focus on functional reconstruction afterward, considering “excursion,” which meant that the graft would be tighter during knee extension. As a result, the femoral tunnel position was proximal to the anatomical position, and the length of the graft was longer than the natural anatomy of ALL.

Previous authors have described the anatomical landmarks and length of ALL, and the mean length of the ALL was reported to vary from 30.41 to 59 mm [1–3, 5–8, 34, 35]. Most authors reported the point of proximal, posterior to LE as the femoral origin, and some reported LE as the origin. In subgroup analysis 1, the anterior position group was more proximal from the LE; thus, the length of the graft increased. In the anterior position group, the mean lengths of the anterior and posterior fiber were 71.4 mm and 69.4 mm, respectively, which was significantly longer than in the anatomical studies and posterior positioned group.

Pearson correlation analysis showed a significant correlation between the anteroposterior femoral position and fiber excursion, meaning that functional reconstruction can be more easily achieved with the posterior positioned tunnel. These results are similar to a previous study by Imbert et al. where the proximal–posterior femoral location was the only position to reveal a decrease in length during knee flexion [26, 36]. If initially located on the anterior side rather than on the LE, the excursion was considered unacceptable and required several repositioning processes. Actually, we required a mean of 3.1 adjustments to find the ideal functional point in the anterior position group, and this value was significantly higher than that noted in the posterior position group. In all, accurately palpating the LE at first and positioning clearly posteriorly compared with the LE can create the anatomical and functional reconstruction and save time.

In this study, 36.3% of the femoral tunnels were located in an anterior position relative to the center of the LE. These findings are similar to those of previous studies on the anatomical variants of ALL femoral origin [2, 6]. Considering the fact that 23–30% of ALLs are directly attached to the LE, a slight anterior positioning relative to the center point of the LE would be acceptable for functional considerations.

For revision ACL reconstruction, the process of finding the ideal femoral tunnel position is challenging. It could be difficult to create a femoral tunnel for the ALL at the desired location because of overlapping tunnels or fixation buttons used in ACL reconstruction, or because of the formation of a new tunnel or an existing tunnel that was widened at the time of revision. In particular, in two cases of revision ACL reconstruction in this study, it was difficult to create a tunnel at the ideal location because the previous femoral tunnel for the ACL reconstruction was positioned too posteriorly and inferiorly. Inevitably, the tunnel position was moved proximally; accordingly, the fiber length became longer. In this study, although not statistically significant, the tunnel position was located more anteriorly and proximally in cases of revision ACL reconstruction. Surgeons should be careful to position the femoral tunnel posterior to the LE during revision ACL reconstruction.

This study has several limitations. First, it had a retrospective design. Second, there were no comparative groups using different surgical techniques. Moreover, in this study, we used the anatomical landmark and finally adjusted it to a functional position. Evaluating the difference in clinical outcome is limited when only the anatomical landmark is targeted. Third, we used a 3D-CT reconstruction model to detect the center point of tunnel, center point of the LE, and length of the fibers; thus, there may have been measurement errors in all the parameters. However, in our study, all the parameters showed good intra- and inter-observer correlation.

## Conclusions

It is challenging to reproduce both anatomical and functional aspects of ALL reconstruction in both primary and revision ACL reconstruction. Especially for functional reconstruction, the femoral tunnel tended to be positioned more proximally than the anatomical position. However, the femoral tunnel position did not affect functional clinical outcomes at the 2-year follow-up.

## Data Availability

The data that support the findings of this study are available upon reasonable request.
